# Application of Nanomaterial-Based Sonodynamic Therapy in Tumor Therapy

**DOI:** 10.3390/pharmaceutics16050603

**Published:** 2024-04-29

**Authors:** Nan Yang, Jianmin Li, Shujie Yu, Guoyu Xia, Dingyang Li, Longlong Yuan, Qingluo Wang, Lijun Ding, Zhongxiong Fan, Jinyao Li

**Affiliations:** School of Pharmaceutical Sciences, Institute of Materia Medica, College of Life Science and Technology, Xinjiang University, Urumqi 830017, China

**Keywords:** sonodynamic therapy, SDT mechanisms, tumor therapy, nanomaterials, combined therapy

## Abstract

Sonodynamic therapy (SDT) has attracted significant attention in recent years as it is an innovative approach to tumor treatment. It involves the utilization of sound waves or ultrasound (US) to activate acoustic sensitizers, enabling targeted drug release for precise tumor treatment. This review aims to provide a comprehensive overview of SDT, encompassing its underlying principles and therapeutic mechanisms, the applications of nanomaterials, and potential synergies with combination therapies. The review begins by introducing the fundamental principle of SDT and delving into the intricate mechanisms through which it facilitates tumor treatment. A detailed analysis is presented, outlining how SDT effectively destroys tumor cells by modulating drug release mechanisms. Subsequently, this review explores the diverse range of nanomaterials utilized in SDT applications and highlights their specific contributions to enhancing treatment outcomes. Furthermore, the potential to combine SDT with other therapeutic modalities such as photothermal therapy (PTT) and chemotherapy is discussed. These combined approaches aim to synergistically improve therapeutic efficacy while mitigating side effects. In conclusion, SDT emerges as a promising frontier in tumor treatment that offers personalized and effective treatment options with the potential to revolutionize patient care. As research progresses, SDT is poised to play a pivotal role in shaping the future landscape of oncology by providing patients with a broader spectrum of efficacious and tailored treatment options.

## 1. Introduction

In recent years, cancer has become one of the most serious health problems globally due to its increasing incidence. However, traditional treatments like chemotherapy, radiotherapy, and surgery can cause significant harm to patients’ bodies and are prone to allowing recurrence and metastasis [1,2]. Therefore, finding safer and more effective ways of treating tumors is a critical goal in today’s medical field. Nanotechnology has attracted considerable attention as an emerging technology in medicine recently. By utilizing the special properties of nanomaterials, precise delivery of drugs can be achieved. As an emerging tumor treatment strategy, SDT has attracted extensive attention and research in recent years [3]. It achieves localized destruction of tumor tissues through the interaction between sound waves and nanomaterials, with advantages such as targeted delivery, minimal trauma, and reusability. Compared with traditional cancer treatments, SDT not only directly destroys tumor cells but also stimulates the body’s immune response and enhances antitumor immune function. This improves treatment effectiveness while reducing damage and side effects on the body.

This review aims to explore the application of nanomaterial-based SDT in tumor therapy. Firstly, it will introduce the fundamental principles of SDT, including its conceptual framework, mechanism of action, and advantages for tumor therapy. Secondly, it will explore the diverse mechanisms through which SDT facilitates tumor treatment. These mechanisms include the induction of immunogenic cell death (ICD), the regulation of the tumor microenvironment (TME), and the modulation of reactive oxygen species (ROS). Subsequently, this review will delve into the utilization of nanomaterials in SDT. Nanomaterials possess unique structures and properties that can generate more robust mechanical and thermal effects when exposed to sound waves, thereby enhancing the therapeutic efficacy of SDT. Indeed, nanomaterials play a crucial role in optimizing the outcomes of SDT in tumor therapy by leveraging their properties. For instance, through surface modification and the functionalization of nanoparticles (NPs), it becomes possible to achieve targeted delivery to tumor tissues, thereby improving treatment selectivity and effectiveness. Additionally, the exploration of SDT in combination with other treatment modalities is a current research focus in the field of tumor treatment. By combining SDT with therapies like chemotherapy, photodynamic therapy (PDT), and immunotherapy, synergistic enhancement of treatment effects can be achieved, ultimately improving the success rate of treatment. However, it is important to conduct comprehensive studies and assess the safety and tolerability of the interactions between different treatment modalities, as their mechanisms of interaction exhibit diversity. To summarize, SDT represents a highly promising and effective emerging tumor treatment strategy [3].

**Figure 1:**
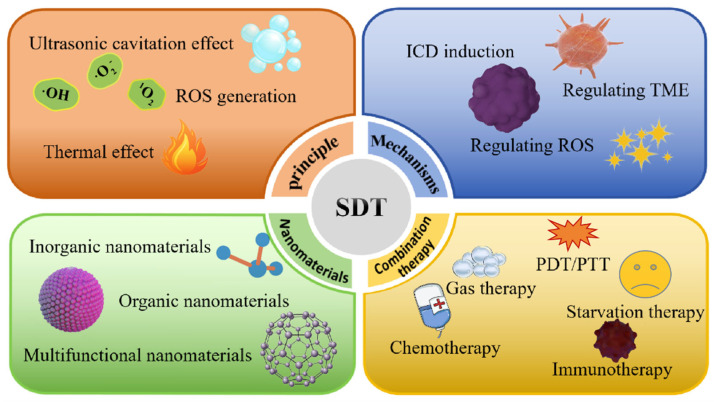
This review comprehensively analyzes and discusses the principles, mechanisms, nanomaterials, and combination therapies involved in SDT.

As shown in Figure 1, this review will comprehensively analyze and discuss the principles, mechanisms, nanomaterials, and combination therapies involved in SDT, thereby providing valuable references and insights for researchers and clinicians in the field of oncology treatment. Through extensive research and practical applications, we are confident that SDT will offer more treatment options and hope for tumor patients, ultimately contributing to the advancement of cancer treatment.

## 2. The Principle of SDT

**Figure 2:**
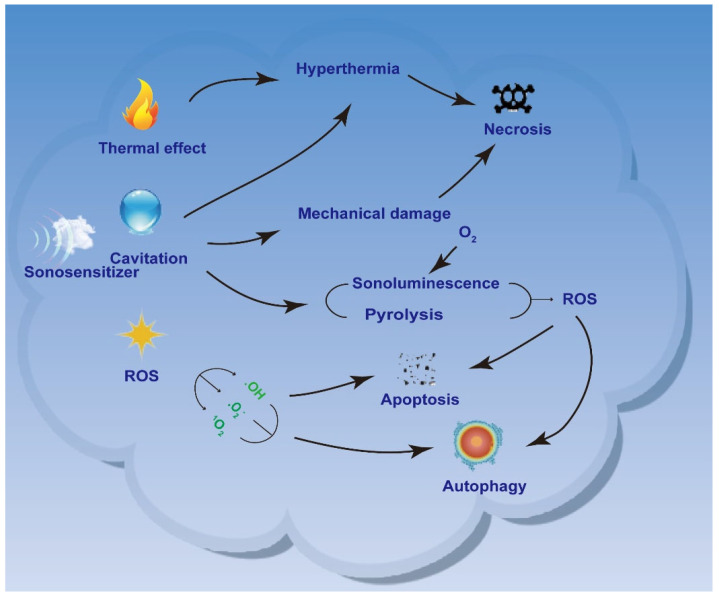
Principle of SDT.

SDT is an emerging, US-based noninvasive treatment for deep solid tumors derived from PDT [4]. US is a non-invasive mechanical wave that can penetrate tissues deeply. Unlike light waves, low-frequency and low-intensity US may have limited ability to damage tumor cells, damaging only a small fraction. Because acoustic sensitizers in tumor tissues are activated by US irradiation, jumping from the ground state to the excited state and generating highly cytotoxic ROS [5,6,7], increasing ROS production can improve the efficiency of SDT [8]. Therefore, US-mediated SDT can kill many tumor cells in the presence of acoustic sensitizers. The properties of US-induced SDT, such as non-invasiveness, superb tissue penetration, and high spatiotemporal control, make it a new therapeutic alternative to conventional tumor therapy. As discussed in the current study, the commonly accepted mechanisms of SDT include ultrasonic cavitation effect, ROS generation, and thermal effects (Figure 2).

The ultrasonic cavitation effect refers to the nucleation, growth, and rupture of US-induced cavitation bubbles in the presence of an acoustic sensitizer. Cavitation effects mainly include inertial cavitation effects and stable cavitation effects [9]. Inertial cavitation effects consist of the formation of cavitation bubbles in liquids under high-acoustic-pressure US and their rapid growth and rupture under the continuous effect of US [10,11]; these effects also generate localized high temperatures and provide sufficient energy for the generation of hydroxyl radicals (·OH). A stable cavitation effect is the periodic oscillation of cavitation bubbles under US irradiation at low acoustic pressure [11], which produces microfluidic jets with low shear force, leading to the flow of the surrounding medium. The cavitation bubble releases a large amount of energy in the process of instant rupture and forms an extreme microenvironment with a temperature of more than 10,000 K and a pressure of more than 81 MPa in the focal area [12]. This process also generates a sonoluminescence (SL) effect, which stimulates acoustic sensitizers to produce ROS, leading to cell damage. Furthermore, SDT produces acoustic-thermal and acoustic-mechanical effects, generating heat in situ and strong shear forces to eliminate surrounding pathological cells. These diverse mechanisms highlight SDT’s potential as a powerful tumor treatment strategy.

SDT can also produce cytotoxic effects by producing ROS, thus killing pathological cells. When SDT is combined with US irradiation, the mechanical action of sound waves and the thermal effect of US may jointly promote the production of ROS, resulting in increased intracellular oxidative stress and eventually cell damage or even apoptosis [13]. ROS can induce the opening of the mitochondrial permeability transition pore (MPTP) by altering mitochondrial membrane permeability and releasing calcium ions from the mitochondria, leading to impaired mitochondrial function, intracellular energy depletion, and ultimately the induction of apoptosis [14]. Secondly, ROS can also oxidize biological macromolecules such as proteins, lipids, and nucleic acids, causing oxidative stress, resulting in an imbalance of oxidation–reduction balance in cells, thus triggering a series of cell-signaling pathways, including the activation of apoptosis signaling pathways [15,16,17]. In addition, high levels of ROS may also lead to oxidative damage to deoxyribonucleic acid (DNA), triggering the activation of intracellular DNA repair mechanisms and apoptosis signaling pathways, ultimately leading to apoptosis [18]. Taken together, the cytotoxicity of US irradiation can be enhanced by utilizing the ROS generated by SDT, which can improve the inactivation rate of tumor cells and thus provide a better therapeutic effect. Based on the current state of research, the two main mechanisms by which SDT induces the generation of ROS are SL [19] and pyrolysis. SL is the light energy generated by the rapid collapse of cavitation bubbles during ultrasonic cavitation. Similar to PDT, SL in SDT stimulates ROS production from acoustic sensitizers via type I and type II responses [20]. There are two ways of generating ROS in SL: One is the excitation of the acoustic sensitizer to generate ROS via reaction with O_2_ or H_2_O, such as single-linear oxygen (^1^O_2_), superoxide anion radicals (O^2−^), or ·OH. The second way is the direct excitation of acoustic sensitizers to produce ROS via ultrasonic cavitation. The ROS produced cause irreversible damage to the cells [21]. Another mechanism by which SDT generates ROS is the direct pyrolysis of water, where acoustic energy causes localized warming of the water, resulting in inertial cavitation. The high-temperature and high-pressure environment created by this cavitation causes water molecules to undergo pyrolysis, producing ·OH and hydrogen atoms. These ROS have an oxidative damage effect on the cells, which results in a therapeutic effect [22].

In addition to acoustic cavitation and ROS generation, US irradiation can induce thermal effects by increasing tissue temperature. In US-mediated therapy, tissue absorption and conversion of mechanical energy from acoustic waves trigger a tissue thermal effect [23]. Thermal effects lead to cell necrosis and eventually cell death. The pyrolysis mechanisms that generate ROS include the high temperatures generated during the implosion of transiently cavitating microbubbles. High temperatures directly decompose water molecules to produce ·OH and hydrogen atoms. These ROS can react with acoustic sensitizers to generate ROS with long half-lives. In addition, acoustic sensitizers can also decompose directly at high temperatures, generating free radicals. These free radicals further react with other endogenous substances within a cell to generate other reactive forms of oxygen, further contributing to cellular damage and therapeutic effects.

## 3. Mechanisms of SDT for the Treatment of Tumors

SDT for tumor treatment is a method of enhancing the effect of therapy by releasing drugs into the tumor tissue. This treatment combines the use of sonic energy with a drug and is designed to increase the concentration of the drug within the tumor cells and enhance its therapeutic effect. In this process, the drug is typically combined with carriers, such as nanoparticles or liposomes, which can release the drug into the tumor tissue in response to the sound waves. Molecular dynamics and other computational methods can be used to study the mechanism of drug release via SDT for tumor treatment [24]. First, a model of the molecular system is needed, including molecules such as drugs, carriers (e.g., nanoparticles), and tumor cells. The structures of these molecules can be obtained from experimental data or computational methods (e.g., quantum chemical calculations). Then, molecular dynamics simulations are used to model the diffusion and release processes of the drug within the carrier, and quantum chemical calculations are used to study the interactions between the drug molecules and the carrier or tumor cells. Next, molecular docking techniques allow prediction of the binding mode and affinity between the drug and target proteins (e.g., receptors on the surfaces of tumor cells). Combined with kinetic simulations, the binding and dissociation processes of drugs with respect to target proteins can be studied to further understand the mechanisms of drug action. Finally, the computational results can be used to guide experimental design to verify the accuracy of the computational model and further validate the drug release mechanism of SDT for tumor treatment [25]. Through the combined application of these computational methods, the drug release mechanism of SDT for tumor treatment can be understood in depth, providing theoretical guidance and prediction for drug design and the optimization of therapeutic regimens. These methods can help researchers better understand a drug’s transport, release, and action mechanisms, thereby improving therapeutic efficacy and reducing side effects. However, the mechanism of SDT for tumor treatment involves several aspects, mainly including the induction of ICD, the regulation of the TME, and the regulation of ROS levels.

### 3.1. Mechanisms of ICD Induction

SDT can not only eliminate tumor cells but also effectively activate antitumor immunity. By utilizing US to selectively activate acoustic sensitizers, SDT induces the production of cytotoxic substances within tumors, ultimately leading to apoptosis and immunogenic death. This process triggers the local release of ICD signals, transforming tumors with low immunogenicity into highly immunogenic ones, thus bolstering the effectiveness of antitumor immunity. Following specific physical or chemical stimulation, tumor cells can release immunogenic signaling molecules, such as heat shock proteins and adenosine triphosphate (ATP), which, in turn, activate antigen-presenting cells (such as dendritic cells), promote T cell activation, and elicit a tumor-specific immune response. This transformation from low to high immunogenicity represents an effective strategy for enhancing antitumor immunity [26,27,28]. Through the stimulation of antitumor immunity, SDT reactivates the host immune response, leading to improved therapeutic outcomes and prognosis. This holds significant importance for enhancing patient prognosis and extending survival. Therefore, SDT not only directly targets and eradicates tumor cells but also boosts the body’s antitumor immunity by inducing the immunogenic death of tumor cells. This presents a novel approach and direction for tumor therapy.

**Figure 3:**
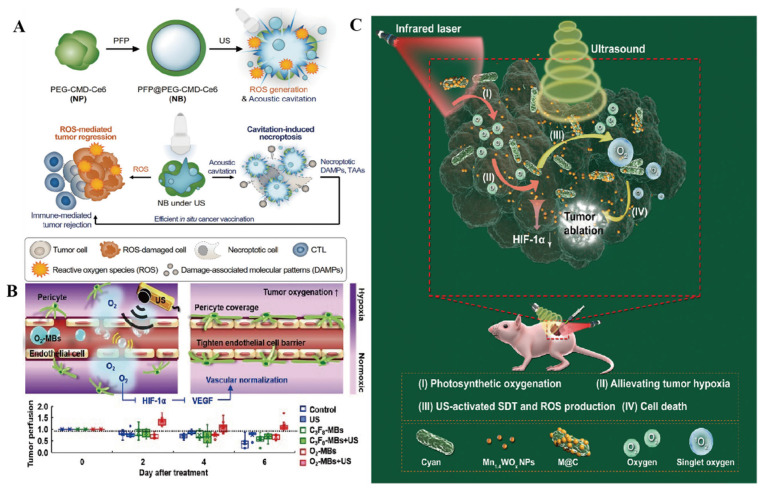
SDT Mechanisms for the treatment of tumors. (**A**) Mechanisms of ICD induction: schematic illustration of necroptosis-inducible NBs for antitumor immune response. Upon US irradiation, NBs generate ROS and induce the cavitation effect. NB-mediated necroptosis provokes release of intact DAMPs, allowing for in situ cancer vaccination. Reproduced with permission from Ref. [30]. Copyright 2020 WILEY-VCH Verlag GmbH & Co. KGaA, Weinheim. (**B**) Concept of US-induced tumor VN using O_2_-MBs. The local oxygen release within tumors during O_2_-MB treatment enhanced oxygenation and inhibited the hypoxia/angiogenesis pathway, allowing VN to be achieved. Reproduced with permission from Ref. [36]. (**C**) Mechanisms for regulating ROS: schematic illustration of M@C for enhancing the SDT efficiency in hypoxic tumors. Oxygen self-contained hybrid sonic sensitizer based on photosynthetic microbial cyanobacteria integrating ultra-small anoxic bimetallic oxide Mn1.4WOx nano-sonar sensitizer referred to as M@C. Reproduced with permission from Ref. [37]. Copyright 2021 Wiley-VCH GmbH.

SDT effectively eradicates tumor cells, triggering the induction and release of tumor-associated antigens (TAAs) and generating damage-associated molecular patterns (DAMPs). These DAMPs include calreticulin (CRT) exposed on the cell surface, high-mobility group protein 1 (HMGB1) secreted from the tumor cells, and ATP and heat shock proteins (HSP70, HSP90) released by the cells [29]. In traditional antitumor therapies such as chemotherapy or radiotherapy, apoptosis in tumor cells often leads to the release of DAMPs. However, these DAMPs may lose their immunogenicity when exposed to the TME, hindering the immune system’s ability to recognize and target tumor cells. To address this challenge, novel tumor immunotherapy strategies have emerged, including immunostimulatory drugs, cellular therapy, and immune checkpoint inhibitors. These approaches serve to activate the immune system and bolster its capacity to attack tumor cells, ultimately leading to improved treatment outcomes. In SDT, physical stimuli such as sound waves and light are utilized to precisely guide drugs for tumor treatment while also triggering cellular immunogenic death events. Unlike conventional antitumor therapies, SDT does not lead to oxidative degradation, allowing for the efficient release of DAMPs without a significant impact. Generally, the outcomes of ICD are directly linked to levels of ROS. ROS play a crucial regulatory role within cells and can activate multiple signaling pathways that induce both cell death and immune responses. Consequently, elevating ROS levels may contribute to promoting immunogenic death events. However, the generation of ICD induced by SDT is not entirely efficient, and the cavitation effect of US can also result in cell membrane fragmentation and the release of immunogenic DAMPs. Ultrasonic cavitation entails the rapid expansion and collapse of small bubbles in a liquid. This phenomenon produces substantial mechanical stimulation, leading to the physical rupture of cell membranes and subsequent release of cell contents, including immunogenic DAMPs. Um et al. developed a nanobubble (NB) composed of a conjugate comprising PEGylated carboxymethyl dextran and chlorin e6 (Ce6) (Figure 3A) [30]. This NB could induce necrotic apoptosis independent of receptor-interacting protein kinase 3 (RIPK3). Upon stimulation via US, the NB effectively facilitated the release of bioactive damage-related molecular patterns by causing burst-mediated disintegration of cell membranes. Consequently, the necrotic apoptosis-induced NBs led to a significant enhancement of antitumor immunity both in vitro and in vivo. This effect was achieved through the maturation of dendritic cells and activation of CD8^+^ cytotoxic T cells. Furthermore, when combined with immune checkpoint blockade, the NBs demonstrated complete regression of primary tumors and exhibited beneficial therapeutic efficacy against metastatic tumors in vivo, as observed in experimental settings.

Studies have shown that ultrasonic cavitation increases the rate of ROS production and induces ICD [31,32]. This effect may stem from the mechanical stimulation and cell membrane rupture triggered by ultrasonic cavitation, leading to the release of ROS and increased cell death. Leveraging ultrasonic cavitation to enhance the immunogenicity of SDT-induced cell death can have three primary effects. Firstly, it aids in the destruction of tumor cells and the release of intracellular antigens, thereby improving antigen recognition by the immune system. Secondly, it influences antigen presentation by tumor cells, resulting in enhanced presentation of antigens to immune cells, which, in turn, boosts the immune response. Lastly, it initiates a local inflammatory response that attracts immune cells to the treated area, further augmenting the immune effect. Moreover, several studies have demonstrated that the cell death induced by US cavitation is more immunogenic [30,33], which enhances the strategy of SDT-induced ICD, turning “cold” tumors into “hot” tumors and strengthening the antitumor immune response [34,35]. This finding provides support for the potential of utilizing US cavitation as a therapeutic modality for tumors and further demonstrates its association with ICDs.

### 3.2. Mechanisms for Regulating TME

The TME refers to the intricate and dynamic environment surrounding tumor cells. It encompasses various components such as cells (including tumor cells), stroma, and signaling molecules. The TME plays a crucial role in tumor development, progression, and response to therapy. Within the TME, there are several key elements, including tumor-associated immune cells, tumor-associated fibroblasts, extracellular matrix, and the vascular system [38]. Tumor-associated immune cells consist of various immune cell populations, such as tumor-infiltrating lymphocytes, macrophages, and myeloid-derived suppressor cells. These immune cells can either promote or inhibit tumor growth and affect a tumor’s response to therapy. Tumor cells constitute a central element within the TME, where their aberrant proliferation and malignant characteristics drive tumor initiation and progression. Various immune cells, including tumor-associated macrophages (TAMs), lymphocytes, and dendritic cells, populate the TME. While capable of triggering antitumor immune responses, these immune cells are also susceptible to tumor-mediated immune evasion strategies, leading to their functional impairment. The TME exerts a profound influence on critical aspects of tumor behavior, including growth, infiltration, and metastasis, and the effectiveness of antitumor therapies. Consequently, comprehending and intervening in the TME constitute a pivotal strategy in cancer treatment, with the goal of enhancing tumor outcomes and ultimately bolstering patient survival rates.

The modulation of the TME through SDT represents a promising avenue in cancer treatment. SDT offers several potential mechanisms for altering the hypoxic TME, such as decreasing oxygen consumption, enhancing endogenous oxygen levels, facilitating the influx of exogenous oxygen carriers into the tumor, and reshaping the TME to counteract hypoxia. These strategies aim to alleviate the hypoxic conditions within the TME, potentially improving treatment efficacy and outcomes in cancer therapy. The elevated metabolic activity and fast growth of tumor cells create a heightened need for oxygen, resulting in hypoxic conditions within tumor tissues. SDT offers a means with which to partially alleviate this hypoxia by directly targeting and destroying tumor cells, thereby easing the burden on the surrounding tissues, and reducing the oxygen demand. Yet, a mere reduction in oxygen consumption is insufficient. SDT also triggers the release of vascular endothelial growth factor (VEGF) via US stimulation, stimulating neo-angiogenesis and enhancing the blood flow to tumor tissues. This process boosts oxygen delivery to the tumors, ultimately raising the oxygen levels within the tumor cells and improving their oxygenation statuses (Figure 3B) [36]. And SDT can be applied in combination with exogenous oxygen carriers, such as oxygen microbubbles [36] and nano-oxygen [39]. These exogenous oxygen carriers can be activated to release oxygen during SDT, providing additional oxygen supply to tumor tissues and ameliorating hypoxia. Gong et al. designed a Cu-CuFe_2_O_4_ nano-enzyme that can alleviate both hypoxia and GSH depletion by continuously catalyzing the generation of O_2_ from H_2_O_2_ in the presence of US, which promotes the production of ^1^O_2_ and thus alleviates hypoxia [40]. 

There is a multifaceted approach to remodeling the TME to counteract hypoxia. SDT plays a crucial role in enhancing the hypoxic TME by disrupting tumor tissue structure, facilitating blood flow and oxygen diffusion through mechanical, thermal, and oxidative stress mechanisms. Moreover, SDT triggers an inflammatory reaction within tumors, activating the immune response, recruiting and stimulating immune cells, and modifying the immunological characteristics of the TME to further reverse hypoxia. Specifically, in US in SDT, mechanical energy is generated to modify the physical and chemical properties of the tumor tissue; this energy includes localized heating and shear forces. This process enhances drug penetration, distribution, and concentration within a tumor, thereby boosting therapeutic effectiveness. By addressing the hypoxic environment within the TME, SDT exerts its antitumor effects through various mechanisms. Firstly, SDT disrupts tumor vasculature, leading to apoptosis, necrosis, and injury to vascular endothelial cells, alleviating tumor hypoxia, reducing internal pressure, enhancing drug permeation, and improving the conditions in the TME [41]. Additionally, SDT impacts the immunological landscape of the TME by releasing TAAs, regulating the activity of tumor-associated immune cells (e.g., TAM and T cells), and modulating the inflammatory response [42]. This process attracts immune cells to the treatment site and influences the levels of inflammatory factors in the TME, indirectly shaping the microenvironment. Lastly, SDT can alter the structure and composition of the tumor extracellular matrix, modify the physical and chemical properties of the TME, and influence the migratory, invasive, and metastatic capabilities of tumor cells. Through these intricate mechanisms, SDT contributes significantly to reshaping the TME and improving therapeutic outcomes in cancer treatment.

### 3.3. Mechanisms for Regulating ROS

In SDT, ROS play a crucial role. These molecules are highly reactive and participate in various biological processes within the cell, including regulating signaling, apoptosis, antimicrobial, and immune processes. The therapeutic effect of SDT largely depends on the production of ROS-mediated oxidative stress. However, if tumor tissues have lower ROS generation and antioxidant overexpression levels, these characteristics may reduce the efficacy of SDT [43,44]. One way to induce ROS production and accumulation is by using US, which can exert a therapeutic effect on tumor cells. Overall, ROS are essential in SDT, and their proper regulation is necessary for attaining optimal therapeutic outcomes.

In SDT, the modulation of ROS activity occurs through several mechanisms. First, SDT can induce US-induced oxidative stress, leading to synergistic effects of drugs and ROS, and it can also alter the internal tumor environment. Specifically, US triggers oxidative stress by activating and inducing the generation of endogenous and exogenous ROS, resulting in cellular damage and death (Figure 3C) [37]. For instance, US activation can stimulate NADPH oxidase and the mitochondrial respiratory chain to increase ROS production. Additionally, US can influence the expression and activity of antioxidant enzymes, thereby regulating the balance between ROS and antioxidant systems. Secondly, SDT can enhance the treatment of tumors by promoting the synergistic effect of drugs and ROS [45]. During SDT, the action of US generates ROS and increases cellular uptake and sensitivity to drugs, resulting in a synergistic therapeutic effect. SDT can improve drug permeability and release by altering the physical and chemical properties of tumor tissues. At the same time, US can also alter the structure and function of cell membranes through mechanical and thermal effects and increase the permeability of cell membranes, thus increasing cellular uptake of exogenous ROS and the release of endogenous ROS [46]. This process amplifies the synergistic effect between the drug and ROS, intensifying damage to tumor cells. Moreover, the mechanical and thermal impacts of US can influence environmental factors within tumor tissues, such as temperature, pH, and oxygen levels. These alterations help regulate ROS production and activity, impacting cell survival and death signaling pathways. Overall, these combined effects exert oxidative stress on tumor cells, leading to cellular damage, apoptosis, and eventual cell death. Consequently, SDT represents a treatment approach that leverages US-induced oxidative stress to combat tumors effectively.

Another strategy employed in SDT for tumor treatment involves reducing intracellular glutathione (GSH) levels. GSH serves as a crucial antioxidant that shields cells from harm by neutralizing detrimental agents like free radicals. However, specific tumor cells exploit GSH to sustain their survival and growth, as elevated GSH levels shield them from oxidative damage induced by ROS. Therefore, in certain scenarios, lowering intracellular GSH levels can serve as an effective approach for treating tumors through SDT [47]. A reduction in intracellular GSH levels can be achieved through two primary pathways: the inhibition of GSH synthesis and the depletion of existing GSH reserves. In cells, the inhibition of the GSH synthesis pathway predominantly relies on reactions facilitated by GSH synthase. By impeding the activity or expression of GSH synthase, the synthesis of intracellular GSH can be hindered, leading to a decrease in GSH levels. For instance, compounds like buthionine sulfoximine (BSO) and γ-glutamyl cysteine synthetase can inhibit the activity of GSH synthase, consequently impeding intracellular GSH synthesis [48]. Another approach to reducing intracellular GSH levels involves depleting the existing pool of GSH molecules. This can be achieved by utilizing specific compounds, such as selenium, which can effectively reduce GSH levels within cells. For example, Chen et al. designed a diselenium-linked dimeric prodrug nanomedicine, FA-SeSe-NPs, in which the diselenium bond responds specifically to ROS, generating selenium radicals to increase ROS levels and reacting with GSH to form S-Se bonds, thereby depleting GSH under conditions of high ROS and GSH levels [49]. These compounds or drugs interact with GSH, rendering it inactive or facilitating its breakdown and metabolism, thus reducing intracellular GSH levels. It is essential to recognize that the impact of intracellular GSH levels on tumor therapy may vary based on factors such as tumor type, cellular environment, and treatment approach.

## 4. Nanomaterials for SDT Realization

In SDT, nanomaterials play a crucial role in enhancing therapeutic outcomes. These nanomaterials typically possess distinct physical, chemical, and biological properties that can serve various functions upon exposure to US stimulation. Commonly used nanomaterials in SDT encompass inorganic, organic, and multifunctional varieties. In the application of nanomaterials, one needs to consider factors such as their preparation methods, surface modifications, and biocompatibility. In addition, issues such as the safety and biological effects of nanomaterials need to be fully investigated and evaluated. The selection and design of different nanomaterials also need to be optimized according to specific therapeutic goals and requirements.

### 4.1. Multifunctional Nanomaterials

The combination of various materials with multiple functionalities is often employed to assemble these nanomaterials. For instance, the merging of metallic NPs with organic nanomaterials can achieve dual effects, encompassing photothermal and acoustic responses, thereby amplifying therapeutic efficacy. The evolution of therapeutic nanomedicines has facilitated the development of multifunctional nano-systems and pioneering disease treatment strategies [50]. This advancement has enabled the multi-functionalization of acoustic sensitizers, leading to improved treatment outcomes. Integration of noble metals, for example, induces surface plasmon resonance effects that enhance the separation of electron holes from the energy band structure [51,52]. Metal–organic frameworks (MOFs) constitute a category of multifunctional nanomaterials. MOFs are crystalline structures composed of metal ions or clusters along with organic ligands. They can serve as drug carriers for controlled drug release upon acoustic stimulation during SDT and for targeted delivery. Additionally, MOFs possess inherent photothermal conversion properties, enabling localized thermal effects under US stimulation, thereby facilitating the destruction of tumor cells and blood vessels. However, the use of MOFs as acoustic sensitizers in SDT for tumor treatment may raise several safety and toxicity concerns, and thus a thorough evaluation of their biocompatibility and toxicity is essential. The biocompatibility of MOF nanomaterials is an important consideration. Although MOF materials are usually highly tunable and chemically functional, they may trigger immune responses or cytotoxicity, especially when MOF nanoparticles enter an organism. Their surface properties may cause cellular damage or inflammatory responses. Therefore, when designing and applying MOF nanomaterials, factors such as their surface modification, solubility, and biocompatibility need to be considered to minimize their adverse effects on organisms [53]. Meanwhile, MOFs may release metal ions or organic ligands, leading to cellular damage or toxic effects [54]. When evaluating the safety and toxicity of an MOF as an acoustic sensitizer for the treatment of tumors via SDT, comprehensive in vitro and in vivo experiments need to be performed and strict regulations and specifications need to be followed to ensure its safe and effective use in clinical practice.

**Figure 4:**
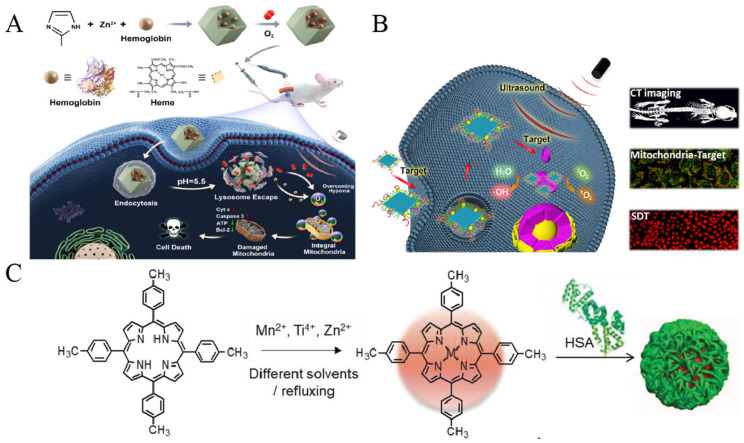
Nanomaterials for SDT realization. (**A**): Synthesis and antitumor mechanism of O_2_@Hb@ZIF-8 NPs. ZIF-8 is constructed of Zn^2+^ ions and 2-methylimidazole ligands. Hb binds to ZIF-8 to form a nanoplatform with potent inhibition of deep tumors under ultrasound irradiation. Reproduced with permission from Ref. [57]. Copyright 2021 American Chemical Society. (**B**): Synthesis process of Au-TiO_2_-A-TPP and a schematic representation of dual-targeted Au-TiO_2_-A-TPP nano-agent for CT-imaging-guided enhanced SDT. Under US irradiation, a large quantity of ROS was produced by Au-TiO_2_-A-TPP and damaged the mitochondria to cause cell apoptosis. Reproduced with permission from Ref. [51]. Copyright 2019 American Chemical Society. (**C**): Synthesis of MTTP complexes and corresponding nanocomplexes with HSA. To obtain MTTP-HSA nanoparticles, MTTP was first dissolved with different solvents/reflux method and then mixed with HSA solution separately by sonication in an ice bath. Reproduced with permission from Ref. [76]. Copyright 2018 WILEY-VCH Verlag GmbH & Co. KGaA, Weinheim.

In recent years, substantial advancements have been achieved in the exploration of MOF materials for cancer therapy [55,56]. For instance, MOFs can serve as carriers for the precise drug delivery of compounds such as chemicals or biomolecules into cancer cells. Yuan et al. designed a Hb@ZIF-8 (HZ) nanoplatform, formed by hemoglobin (Hb), composed of natural metalloporphyrin and zeolite imidazolium ester backbone 8 (ZIF-8) [57], which showed a tumor-inhibitory effect under US (Figure 4A). Hb acts as an efficient and safe oxygen carrier, rendering the NPs biocompatible and a plentiful source of oxygen. ZIF-8, a MOF material known for its high stability and controllability, exhibits excellent drug-carrying properties and safeguards Hb from degradation until it reaches the TME, thereby enhancing the efficacy of SDT. Zhong et al. developed a nanotherapeutic formulation (DOX/Ce6@ZIF-8@PDA) incorporating polydopamine (PDA) encapsulated with ZIF-8, Ce6, and the anticancer drug doxorubicin (DOX) [58]. By utilizing ZIF-8 as a carrier, both Ce6 and DOX were delivered to facilitate a synergistic effect between SDT and chemotherapy. The presence of PDA enhanced interactions with the cell membrane, facilitating endocytosis of the platform and enabling more precise entry of drug carriers into the intracellular space. This mechanism ultimately led to increased intracellular drug concentrations and enhanced lethality against cancer cells. The efficacy of this therapeutic platform in inhibiting tumor growth was demonstrated through both in vivo and in vitro experiments. Similarly, Zhao et al. prepared a drug-carrying system consisting of ZIF-8 loaded with Ce6 and 2-dodecyl-6-methoxycyclohexa-2,5-diene-1,4-dione (DMDD) [59]. It was wrapped using a bionic cell membrane, finally yielding ZIF-8@DMDD/Ce6@cytomembrane (ZDC@M) NPs. ZDC@M improved the ability of the drug to penetrate the cell membrane and release the drug deep into the tumor cells, thus enhancing its ability to kill tumor cells. Through in vivo and in vitro experiments, researchers observed that ZDC@M exhibited favorable biocompatibility and safety profiles, along with significant tumor suppression effects, demonstrated in both in vivo and in vitro settings.

MOFs can also be harnessed for the creation of acoustic sensitizers capable of converting acoustic energy into either thermal or mechanical energy. In the context of SDT, MOF materials are adept at absorbing acoustic energy and transforming it into localized thermal energy. This process induces a thermal effect that facilitates enhanced drug penetration and targeted destruction of tumor cells. Zhang et al. constructed a nanoplatform for loading an alkyl radical generator, 2,2-azobis[2-(2-imidazolin-2-yl)propane] dihydrochloride (AIPH), onto a zirconium MOF (Zr-MOF) [60]. The Zr-MOF@AIPH NPs have the capability to generate singlet oxygen and alkyl radicals, effectively targeting and eliminating tumor cells even under normoxic and hypoxic conditions. Furthermore, the decomposition of AIPH leads to the generation of nitrogen, which can reduce the cavitation threshold and enhance the acoustic cavitation effect, thereby improving the therapeutic efficacy of SDT. Results from both in vitro and in vivo experiments demonstrated the promising antitumor effects and biocompatibility of Zr-MOF@AIPH. While MOFs hold significant potential for SDT applications, there exist limitations related to the charge transfer efficiency between ligands and metal ions within some MOF materials, impacting the overall therapeutic effectiveness of SDT. This limitation primarily revolves around the charge transfer and photothermal conversion properties of MOF materials. Researchers are actively exploring various strategies for addressing this challenge. One approach involves optimizing the interaction between metal ions or clusters and organic ligands by adjusting the structure and design of MOF materials to enhance charge transfer efficiency. Another strategy entails introducing external adjuncts, such as auxiliary photosensitizers or additional metals, to boost the photothermal conversion efficiency of MOF materials. Meng et al. engineered a MIL@Ag heterostructure featuring silver NPs (Ag NPs) assembled in situ on its surface to serve as an acoustic sensitizer [61]. This structure was further encapsulated in polyethylene glycol (PEG) to create MIL@Ag-PEG nanomaterials. These nanomaterials demonstrated remarkable efficiency in separating electron–hole pairs and generating ROS. Through the incorporation of Ag NPs, the nanomaterials exhibited a significant enhancement in electron transfer efficiency and ROS generation capability when subjected to US stimulation. Studies of in vitro and in vivo antitumor activity and in vitro biocompatibility showed that MIL@Ag-PEG excelled in terms of ROS generation efficiency, electron transfer efficiency, and oxygen adsorption capacity. Consequently, these enhancements culminated in an amplified SDT effect. The findings from these studies offer valuable insights and a solid theoretical foundation for the advancement of innovative and potent cancer therapy drugs.

### 4.2. Inorganic Nanomaterials

Inorganic nanomaterials, such as gold, silica, and iron oxide, have emerged as valuable components for SDT. These materials boast high photothermal conversion efficiency and exceptional acoustic response properties, enabling them to induce a localized thermal effect when stimulated by US. This localized thermal effect can effectively destroy tumor cells and blood vessels through either thermal conductivity or photothermal conversion. Moreover, these inorganic nanomaterials can serve as effective carriers for drug delivery, releasing therapeutic agents upon US stimulation. The utilization of inorganic acoustic sensitizers holds great promise for SDT applications owing to their high stability and prolonged cycle times. The remarkable stability of these inorganic acoustic sensitizers allows them to remain functionally stable within the body for extended periods without substantial degradation or inactivation. This characteristic ensures that they maintain their acoustic sensitizing activity during circulation, thereby enhancing the durability of the therapeutic effect. Furthermore, the extended circulation time of inorganic acoustic sensitizers is a pivotal factor contributing to their potential application. Prolonged circulation enables these sensitizers to remain in contact with tumor cells or blood vessels for an extended duration, thereby amplifying the therapeutic impact of sound waves. Additionally, prolonged circulation facilitates the accumulation of inorganic acoustic sensitizers within tumor tissues in vivo, minimizing the impact on healthy tissues. This cumulative effect serves to bolster the overall therapeutic efficacy of SDT. However, the biocompatibility of inorganic nanomaterials is an important issue. Although many inorganic nanomaterials have shown good biocompatibility in in vitro studies, they may trigger immune reactions or cytotoxicity in vivo. Therefore, inorganic nanomaterials must be thoroughly evaluated for biocompatibility induced in vivo, including in relation to cytotoxicity testing, immunological reactions, and inflammatory responses. They may accumulate in vivo in specific organs or tissues, leading to potential toxic effects [62]. Therefore, factors such as their surface modification, solubility, and biocompatibility need to be considered when designing and applying inorganic nanomaterials to minimize their adverse effects on organisms. Also, metal ions or organic ligands that may be released from inorganic nanomaterials need to be closely monitored to avoid triggering cellular damage or toxic effects.

Inorganic nanomaterials can be classified into metallic and non-metallic categories. Metallic inorganic materials typically encompass metal oxides (such as titanium dioxide and zirconium dioxide), metal sulfides (like molybdenum disulfide and copper disulfide), and metal NPs (including gold NPs and Ag NPs). These metallic materials are renowned for their favorable optical, electrochemical, and catalytic properties, rendering them invaluable in the realm of acoustic sensitizers. They serve to augment the acoustic cavitation effect and facilitate the generation of free radicals or ROS, contributing significantly to the efficacy of therapeutic interventions. Harada et al. discovered that TiO_2_ NPs exhibited a cytotoxic effect on tumor cells when exposed to US [63]. Subsequently, TiO_2_ NPs have garnered widespread attention as prominent acoustic sensitizers [64]. However, standalone TiO_2_ acoustic sensitizers have certain limitations, including inadequate stability and specificity. To address this issue, Cao et al. initially synthesized TiO_2_ nanosheets (NSs) with highly exposed (001) facets, onto which Au nanocrystals were selectively grown at the edges to create a SDT-enhanced acoustic sensitizer known as Au-TiO_2_ NSs (Figure 4B) [51]. The ROS generation efficiency of Au-TiO_2_ NSs surpassed that of pure TiO_2_ NPs. Furthermore, the researchers modified Au-TiO_2_ NSs with mitochondria-targeted triphenylphosphine (TPP) and AS1411 aptamers to fashion a mitochondria-targeted acoustic sensitizer (Au-TiO_2_-A-TPP). Through in vitro and in vivo experiments, it was demonstrated that Au-TiO_2_-A-TPP could generate a substantial quantity of ROS and effectively impede tumor growth with notable SDT efficiency [51]. In a separate study, Ning et al. prepared a nano-system (C-TiO_2_/TPZ@CM) for SDT consisting of cancer cell membranes encapsulated with C-TiO_2_ hollow nanoshells (HNSs) containing tirapamide (TPZ) [65]. This nano-system generates reactive ROS in the presence of US and has been proven in ex vivo experiments to have good biosafety and drug loading capacity for effectively killing tumor cells.

Non-metallic inorganic nanomaterials encompass a variety of materials, including carbon-based materials, semiconductor materials, and nitride materials. These materials have unique electronic structures and optical properties that make them suitable for applications such as photoacoustic imaging, PDT, and acoustic power therapy. In the field of inorganic semiconductor materials, silicon nanostructures have been identified as valuable tools in SDT when exposed to US [66]. Sun et al. employed platinum nanomaterials to enhance the photothermal effect in SDT by modifying silicon nanowires [67]. Through an in situ reduction method, they developed Si-Pt nanocomposites and observed that these composites could generate a substantial quantity of ROS when exposed to US, thus serving as effective SDT acoustic sensitizers. Within the TME, Si-Pt NCs demonstrated the capacity to convert excess hydrogen peroxide into ROS and exhibited promising chemokinetic therapeutic activity. The uniform size and distribution of Pt NPs rendered the Si-Pt NCs superior to pure Pt NPs for SDT. In both in vivo and in vitro experiments, the composites, when subjected to US irradiation, produced the highest levels of ROS and significantly hindered the growth of tumor cells. Furthermore, graphene-based materials have been widely utilized in tumor therapy [68] and demonstrate significant tissue penetration when combined with SDT. Lee et al. presented a therapeutic approach for combating metastatic ovarian cancer spheroids involving the use of PEG and Ce6-functionalized graphene nanoribbons (GNRs) for targeted delivery [69]. They found that GNR-PEG exhibited superior cytocompatibility and more effectively blocked tumor spheroid adhesion compared to GO-PEG. Notably, GNR-PEG demonstrated a longer-lasting adhesion-blocking effect, surpassing conventional antibody-blocking methods in effectiveness. Moreover, Ce6-loaded GNR-PEG (GNR-PEG-Ce6) proved capable of eradicating ovarian cancer spheres through SDT. However, the swift recombination of acoustically excited electron–hole pairs in graphene materials posed a significant limitation to their application in SDT. In a related study by Wang et al. [70], an efficient nano-acoustic sensitizer system was proposed to enhance SDT. This system was constructed using reduced graphene oxide (rGO) NSs bridged with zinc oxide and gold NPs and coated with polyvinylpyrrolidone (PVP). Under US irradiation, the ZnO NPs facilitated the generation of separated electron–hole pairs, while the narrow bandgap of graphene oxide NSs facilitated the transfer of electrons from ZnO to Au NPs. This process effectively inhibited the recombination of electron–hole pairs and significantly elevated the production of ROS, leading to the destruction of cancer cells.

### 4.3. Organic Nanomaterials

Polymer NPs and lipid NPs are extensively utilized in SDT as organic nanomaterials. These nanomaterials serve as carriers for drugs, enabling controlled release upon US stimulation and enhancing the local drug concentration within tumor tissues. Additionally, they can induce mechanical effects through US stimulation and thus destroy tumor cells and blood vessels. Organic acoustic sensitizers have several advantages over inorganic materials, including well-defined chemical structures, controlled synthesis processes, good biodegradability, and high generation of ROS [8,71,72]. However, they also face certain challenges, such as poor water solubility, high phototoxicity, and limited tumor tissue targeting ability [62]. Recent studies have focused on improving the performance of organic acoustic sensitizers to enhance their potential applications in SDT.

By loading hydrophobic acoustic sensitizers onto nanocarriers, their water solubility can be significantly improved, facilitating enhanced accumulation in tumor tissues through Enhanced Permeability and Retention (EPR) effects [73]. Human serum albumin (HSA) serves as an immunogenic, highly water-soluble, and biocompatible natural carrier for various hydrophobic molecules. HSA NPs have the capability to accumulate in tumor tissues via the EPR effect. Furthermore, the presence of the albumin receptor on the surfaces of tumor cells can further boost the intracellular uptake efficiency of acoustic sensitizers carried by HSAs [74,75]. Ma et al. designed a novel acoustic sensitizer for SDT [76]. They formed three metalloporphyrin complexes, collectively called MTTP, by chelating 4-methylphenylporphyrin (TTP) with different metal centers and encapsulated it with HSA to form MTTP-HSA NPs (Figure 4C). Due to the high reactivity and short half-life of O_2_, the organelle-targeting ability of chemo-acoustic sensitizers is a key determinant of their therapeutic efficacy [77]. In hypoxic environments, the SDT effect of chemical acoustic sensitizers is limited because there are not enough oxygen molecules in the hypoxic environment to participate in the therapeutic response. Therefore, Ma et al. coupled TPP with cholesterol (Chol) and subsequently with Hematoporphyrin Monomethyl Ether (HMME) through strong hydrophobic interactions with liposome assembly to form TTP-Lipo-HMME NPs, which, in turn, target mitochondria, rapidly releasing HMME under US irradiation and thus producing ^1^O_2_, which, in turn, induces cell death [78]. Researchers are improving the organelle-targeting ability of chemo-acoustic sensitizers by improving their therapeutic efficacy in hypoxic environments, e.g., by utilizing targeted NPs as drug carriers, or by developing novel chemo-acoustic sensitizers. Perfluoro hexane (PFH) has been widely studied and used in SDT as a hydrophobic organic liquid compound. PFH has excellent stability and oxygen-solvent properties, allowing it to efficiently absorb ambient oxygen and exhibit a unique ability to change phases when stimulated by US. When PFH is stimulated by US, tiny bubbles inside it aggregate, forming a large bubble and releasing adsorbed oxygen. This phase transition process can be repeated, making PFH an O_2_ carrier capable of controlled oxygen release [79,80]. Zhang et al. successfully synthesized hollow mesoporous Prussian blue (HPB) NPs and co-loaded them with HMME and oxygen-saturated PFH [81]. Subsequently, these NPs were encapsulated in red blood cell (RBC) membranes to form RBC-HPBS-HMME-PFH complexes with US-responsive drug- and O_2_-releasing capabilities for SDT and US imaging. Due to the enhanced permeability and EPR effect and the excellent transmembrane and diffusion ability of erythrocytes, the accumulation and uptake of RBC-HPBS-HMME-PFH in tumor tissues and cells was enhanced. In the presence of US, the RBC membrane is disrupted, resulting in the release of HMME and O_2_, and this complex significantly improves the performance of SDT and US imaging compared to that for NPs saturated without oxygen-saturated PFH.

## 5. Nanomaterial-Based SDT Combination Therapy

Nanomaterial-based SDT combination therapy is a strategy in which SDT is integrated with other therapeutic approaches to enhance treatment efficacy and broaden the scope of treatment. This approach harnesses the diverse properties of nanomaterials to improve therapeutic effectiveness while minimizing side effects, offering a more potent treatment option for challenging medical conditions. In the present study, it has been demonstrated that combining SDT with PDT, PTT, gas therapy, chemotherapy, starvation therapy, and immunotherapy can enhance the efficiency of treating malignant tumors [82,83,84,85].

### 5.1. Shortcomings and Challenges of SDT

Despite the several advantages of SDT in the treatment of tumors, including non-invasiveness, an excellent local therapeutic effect, the ability to reduce drug doses and side effects, and potential multidisciplinary applications, it still faces a few challenges. SDT still requires more improvement than other established oncology therapies, such as chemotherapy, radiotherapy, and immunotherapy. First, SDT requires more effective therapeutic approaches and techniques, especially with regard to its limited therapeutic effects against deep-seated tumors [86]. Second, SDT requires more selective and targeted therapies. Current SDT technologies face challenges in targeting tumor tissues, so there is a need to develop novel acoustic-dynamic therapeutic agents that can accurately identify and target tumor cells. In addition, the biosafety and toxicities of SDT need to be further researched and improved. As mentioned earlier, acoustic sensitizers may have adverse effects on surrounding normal tissues, and thus their biocompatibility and toxicities need to be evaluated through clinical trials and animal model studies, and appropriate safety measures and treatment protocols need to be developed. In conclusion, although SDT has potential in the field of tumor therapy, further improvements are still needed to enhance its therapeutic efficacy and safety.

As a promising tumor therapy, SDT faces several challenges: tumor heterogeneity, variations in ultrasound penetration across tissues, and ensuring treatment consistency. Tumors are highly heterogeneous, with different parts exhibiting diverse biological properties and treatment responses. This complexity complicates SDT delivery, potentially compromising treatment comprehensiveness and efficacy. Addressing these challenges requires further research and technological advancements, such as improving ultrasound penetration and precision and developing targeted therapies [87]. Second, there are differences in the ability of US to penetrate in different types of tissues. For example, skeletal, adipose, and muscular tissues have different penetrative qualities for ultrasound, which may affect the effectiveness of SDT. For deep tumors, higher energy may be required to allow US to penetrate through to the target area, which may increase the risk of damage to surrounding normal tissues [88]. Finally, ensuring consistency of SDT in practice is a challenge. This involves a number of aspects, including the precision of the ultrasound delivery and focusing technique, the delivery and distribution of the acoustic sensitizer, and the monitoring and regulation of the treatment process. Failure to achieve consistency in these areas can lead to uncertainty and fluctuations in treatment outcomes, thereby compromising the reliability and efficacy of a treatment. Further research and technological development are needed to address these challenges and limitations.

### 5.2. Combined SDT and PDT/PTT Treatment

The combination of SDT with PDT or PTT offers a comprehensive treatment approach that leverages the synergy between sound waves, photothermal effects, and photosensitizers, thereby enhancing therapeutic outcomes and broadening treatment possibilities. In this combined therapy, SDT induces the formation and collapse of microbubbles under the influence of sound waves, creating mechanical stress and localized temperature elevations to disrupt tumor cell structure and function. On the other hand, PDT and PTT involve activating photosensitizers using light or heat energy, leading to oxidative stress or localized temperature elevations, thereby initiating photothermal or photochemical reactions for tumor cell destruction. The key advantage of this combination therapy lies in its ability to amplify therapeutic effects by engaging different mechanisms and pathways of action, resulting in a more comprehensive and effective treatment regimen. Moreover, combining SDT with PDT/PTT can mitigate damage to normal tissues associated with monotherapy, enhancing the safety and tolerability of the overall treatment approach.

**Figure 5:**
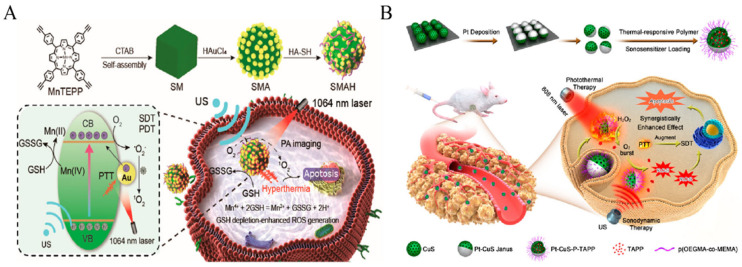
Combined SDT and PDT/PTT treatment. (**A**) Schematic of near-infrared II-activatable self-assembled manganese porphyrin–gold heterostructures for photoacoustic-imaging-guided acoustic power-enhanced photothermal/photodynamic therapy. Reproduced with permission from Ref. [93]. Copyright 2023 American Chemical Society. (**B**) Synthesis and antitumor mechanism of PCPT. Reproduced with permission from Ref. [94]. Copy-right 2019 American Chemical Society.

PDT generates ROS when exposed to near-infrared (NIR) light, and the use of NIR-responsive photosensitizers can improve its penetration depth. However, PDT has certain limitations when it comes to treating deep-seated tumors [89]. On the other hand, NIR-mediated PTT shows promise as an effective antitumor approach [90,91,92]. PTT utilizes NIR light energy to activate a photothermotransduction agent (PTA), which generates heat rapidly for therapeutic purposes, leading to cancer cell death. Therefore, combining PTT with SDT becomes necessary to overcome these limitations and optimize treatment outcomes. Xu et al. aimed to enhance the efficacy of SDT and PDT for tumor treatment (Figure 5A) [93]. They first facilitated the self-assembly of manganese porphyrin NPs (MnTEPP) to synthesize manganese porphyrin (SM) NPs. Subsequently, by attaching the porphyrin terminal alkynyl group, they initiated the in situ growth of gold NPs on the surface of SM NPs, resulting in the formation of plasmonic SMA heterostructures. Further modifications involving hyaluronic acid (HA) led to the synthesis of water-soluble and targeted SMAH nanocomplexes. This nanocomplex can provide thermotherapy and large amounts of ^1^O_2_ via NIR-II light and US irradiation, which can be used to kill tumor cells. In the cited study, the synergistic therapeutic effect of SDT/PDT/PTT was improved by increasing the yield of ^1^O_2_. Furthermore, Liang et al. designed Pt-CuS NPs with good water solubility and biocompatibility (Figure 5B) [94]. These NPs contain a hollow semiconductor, CuS, for the loading of the acoustic sensitizer (tetra-(4-aminophenyl) porphyrin, TAPP), thus realizing SDT. Pt-CuS NPs can act as photothermal converters under 808 nm laser irradiation, and the deposition of Pt has nano-enzymatic activity that increases SDT-induced production of highly toxic ROS, thus promoting apoptosis. Thus, the synergistic effect of SDT and PTT can efficiently kill tumor cells and regulate cancer therapy.

### 5.3. Combination of SDT and Gas Therapy

Gas therapy is a less-harmful, non-resistant, and shorter-course cancer treatment strategy, and there has been much interest in the study of gas delivery [95]. The gaseous transmitters used (e.g., NO, CO_2_, and O_2_) can act as signaling molecules that mediate signaling pathways and be used to treat cancer [96]. The combination of gas therapy and SDT is selective in killing tumor cells while protecting normal tissues [97] and has the advantages of non-invasiveness and high penetration regarding US and high efficacy and biosafety in terms of gas, so this combination treatment option has attracted widespread attention [98,99].

Gas therapy synergistically enhances SDT mainly through the direct killing effect of gases [100], the promotion of drug release and deep penetration [101], and antitumor immune responses [102]. An et al. designed a bionic nanoplatform that combines NO gas therapy with SDT [103]. The designed nanoplatform (GCZ@M), with dual pH/ultrasonic responsiveness, homologous targeting, and low phototoxicity, consists of zeolite imidazolium backbone-8 material loaded with Ce6 and NO-generating agent (S-Nitrosoglutathione, GSNO) encapsulated with homozygous carcinoma cell membranes. Guided by the homologous targeting ability of the tumor cell membrane, GCZ@M can accumulate continuously at the tumor site. In an acidic environment, GCZ@M undergoes degradation and sustained release of encapsulated Ce6 and GSNO, and the degradation of GCZ@M is accelerated under US stimulation. Meanwhile, NO produced by GSNO and ROS produced by Ce6 interact, generating highly cytotoxic peroxynitrite (ONOO), which enhances the inhibitory effect on tumor growth. In addition, repeated irradiation with US accelerates tumor blood flow and increases O_2_ levels at the tumor site, thereby alleviating the hypoxic environment of the tumor and improving the therapeutic efficacy of SDT. Based on the results of in vivo and in vitro experiments, combined air–acoustic power therapy can effectively eliminate tumors. In addition to this, Yin et al. prepared a nano-sonar sensitizer, MnPcS@HPO, for O_2_ self-supplementation for SDT enhancement [104]. They first hybridized HSA to Hb via disulfide bonds and then loaded manganese phthalocyanine (MnPcS) into the hybridized protein to form MnPcS@HPO. Based on in vitro and in vivo experiments, the nano-systems were able to target the tumor site and produce ^1^O_2_, thereby killing tumor cells, and the nano-systems were biosafe.

### 5.4. Combined Treatment with SDT and Chemotherapy

Combining SDT with chemotherapy represents a valuable therapeutic strategy that not only enhances treatment efficacy but also mitigates treatment-related side effects. This approach capitalizes on the mechanical effects induced by US and the chemical actions of chemotherapeutic agents, synergistically improving overall therapeutic outcomes. By integrating SDT with chemotherapy, a complementary effect can be achieved, maximizing efficacy while minimizing adverse effects. Studies indicate that the combination of SDT and chemotherapy can lead to enhanced drug penetration and absorption within tumor cells, thereby augmenting the effectiveness of chemotherapy. This combined approach offers a promising avenue for optimizing cancer treatment protocols and improving patient outcomes [105,106].

**Figure 6:**
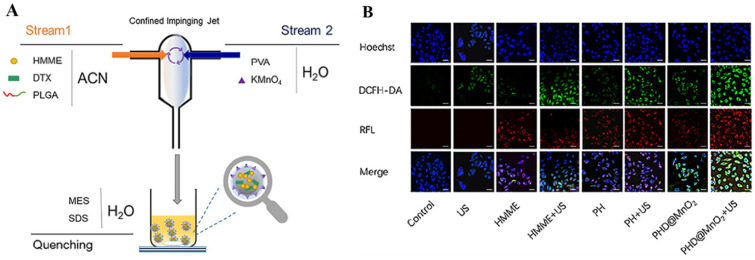
Combined treatment with SDT and chemotherapy. (**A**): One-step preparation of PLGA-HMME-DTX@MnO_2_ NPs via flash nanoprecipitation. (**B**): Cellular ROS generation in MCF-7 cells. The white scale bars represent 25 μm Reproduced with permission from Ref. [108].

Zhang et al. developed a nanoplatform to enhance the effectiveness of SDT and chemotherapy [107]. They created HSA/IR780/PIC NPs (HIP NPs) through self-assembly using HAS, IR780, and Piceatannol (PIC). This innovative combination utilized HSA as a biocompatible carrier, IR780 as an acoustic sensitizer and imaging probe, and PIC as a potent inhibitor of HIF-1α (a transcription factor associated with tumor hypoxia) and a chemotherapeutic agent. In the presence of US, HIP NPs generated ROS while releasing PIC, inhibiting HIF-1α expression and exerting chemotherapeutic effects. By reducing the expression level of HIF-1α, the therapeutic effect of SDT was enhanced. In vivo and in vitro experiments demonstrated that HIP NPs exhibited stronger tumor enrichment ability, excellent imaging performance, lower systemic toxicity, and the ability to overcome SDT resistance caused by hypoxia. These findings indicated that the nano-systems achieved a combined effect of chemotherapy and SDT. It is worth noting that tumor hypoxia, reductive microenvironments, and high levels of GSH pose significant challenges when combining SDT and chemotherapy. Cao et al. developed a multifunctional metal-hybridized nano-system aimed at enhancing the efficiency of antitumor therapy by combining chemotherapy and SDT (Figure 6A) [108]. They first prepared a nanoparticle (PHD) carrying docetaxel (DTX) and the acoustic sensitizer HMME using polylactic-co-glycolic acid (PLGA). Subsequently, the researchers deposited the hybridized metal MnO_2_ on the PHD to form a multi-functional metal hybrid nano-system (PHD@MnO_2_). The in vivo and in vitro results demonstrated that PHD@MnO_2_ generated more ROS and reduced GSH levels under the influence of US (Figure 6B). Additionally, the nano-system exhibited good biocompatibility and biosafety. Moreover, PHD@MnO_2_ effectively overcame tumor tissue hypoxia and improved the efficacy of combined chemotherapy and SDT. This innovative approach developed by Cao et al. showcases the potential of PHD@MnO_2_ in overcoming challenges associated with tumor treatment, particularly in synergizing the effects of chemotherapy and SDT for improving anti-cancer efficacy.

In the context of SDT, Pt nano-enzymes have emerged as a promising tool for enhancing treatment efficacy. These nano-enzymes possess the ability to catalyze the production of oxygen from hydrogen peroxide, thereby alleviating the hypoxic state of tumor tissues. The generated oxygen not only ameliorates the hypoxic environment but also serves as a substrate for US-induced ROS production. Furthermore, Pt nano-enzymes can generate highly toxic ·OH radicals, which directly exert a lethal effect on tumor cells. These unique properties make Pt nano-enzymes highly valuable for enhancing the sensitivity of SDT. Through their actions, Pt nano-enzymes facilitate the supply of oxygen to hypoxic tumor tissues while simultaneously inducing direct tumor cell death. Consequently, the effectiveness of tumor treatment is improved. These characteristics position Pt nano-enzymes as highly promising nanomaterials for SDT applications [109]. Additionally, paclitaxel (PTX), a widely utilized clinical chemotherapeutic agent, has been prominently integrated into SDT combination therapy. PTX is derived from the bark of the redbud tree and serves as a broad-spectrum antitumor drug. Its primary mode of action involves the inhibition of microtubules and the induction of apoptosis in cancer cells [110]. The combined administration of PTX and SDT represents a multifaceted approach to targeting tumors, thereby enhancing therapeutic outcomes through complementary mechanisms. By leveraging the synergistic properties of Pt nano-enzymes and the antitumor effects of PTX, the combined application of SDT can effectively orchestrate interventions against tumors. This includes alleviating hypoxia, augmenting oxidative stress, and triggering apoptosis in tumor cells, thereby elevating the sensitivity and overall effectiveness of the treatment strategy. Therefore, Zhao et al. used Pt nano-enzymes (DC@Pt) encapsulated in stabilized micelles made from prodrug DP and DC and modified with homologous tumor cell membranes (DPC@Pt@M) to create redox-reactive nanoplatforms [109]. The prodrug micelles (DC and DP) were generated by conjugating DSeDP-alt-PEG containing multiple diselenide bonds to Ce6/PTX. Pt nanoenzymes were loaded into DC to form stable micelles (DC@Pt). A biomimetic nanoplatform based on oxidation/reduction reactions was created by modifying nano-micelles (DPC@Pt@M) with homologous tumor cell membranes, enabling the combined treatment of colon cancer with SDT and chemotherapy.

### 5.5. Combined SDT and Starvation Therapy

Starvation therapy functions by impeding the growth and division of tumor cells through restricting their nutrient intake. Tumor cells predominantly metabolize sugar via the aerobic oxidation pathway in mitochondria and the anaerobic glycolysis pathway in the cytoplasm. Regardless, when subjected to US stimulation, acoustic sensitizers with mitochondria-targeting capabilities can produce ROS to disrupt the mitochondria, thereby inhibiting a portion of the energy supply. Nevertheless, tumor cells can still derive energy from anaerobic glycolysis in the cytoplasm, enabling them to sustain their metabolic needs even under the influence of starvation therapy [111]. Zhang et al. prepared a core/shell structured PLGA NPs for synergistic SDT and starvation therapy [112]. The core of these NPs was wrapped with glucose oxidase (GOx), which oxidizes glucose to gluconic acid in the presence of oxygen. In addition, IR780 and HMME were wrapped around the surfaces of these NPs for SDT. When PLGA NPs enter the tumor vasculature, they can effectively accumulate into the tumor tissue and diffuse into the deep tumor via the mitochondrial targeting of IR780. In the presence of US, the production of excessive ROS by HMME can induce apoptosis in cancer cells. Meanwhile, Gox blocks the supply of glucose and inhibits anaerobic glycolysis in the cytoplasm, thus further inhibiting the growth of malignant tumors. This synergistic treatment method consisting of SDT and starvation therapy showed good therapeutic effects in the experiment.

In starvation therapy, the use of GOx indeed contributes to the intensification of the hypoxic TME by converting O_2_ into H_2_O_2_. However, this exacerbation of the hypoxic TME can potentially diminish the effectiveness of SDT. To overcome this challenge, researchers have employed various strategies for enhancing the therapeutic efficacy of SDT. One such strategy involves utilizing materials like MnO_2_ as a carrier to degrade H_2_O_2_ and generate O_2_. This process alleviates the hypoxic TME and ensures an adequate oxygen supply for acoustic radiation during SDT. Therefore, Zhang et al. successfully constructed a cascade catalytic nanoplatform (GOx-MnO_2_/HMME) by combining mesoporous MnO_2_ NPs, HMME, and GOx, realizing starvation therapy and SDT simultaneously [113]. Specifically, GOx catalyzed the oxidation of glucose, thus producing gluconic acid and H_2_O_2_, whereas MnO_2_ NPs decomposed H_2_O_2_ into O_2_, thereby alleviating the extent of hypoxic TME. This cascade catalytic nanoplatform was designed not only to effectively consume glucose for starvation therapy but also to enhance ROS production for HMME-mediated SDT effects. In this way, the therapeutic effect of SDT in hypoxic TME environments can be enhanced. The advantage of this approach is that through the cascade catalytic reaction, it can address both the hypoxia problem caused by starvation therapy and the reduced effectiveness of SDT therapy, thus improving therapeutic efficacy.

Furthermore, glyceraldehyde-3-phosphate dehydrogenase (GAPDH) stands as a pivotal enzyme in the glycolytic pathway [114,115,116]. It participates in the conversion of glucose into pyruvate and phosphoglyceraldehyde while also contributing to ATP production. Under normal circumstances, GAPDH plays a crucial role in cellular energy metabolism. However, in specific conditions such as hypoxia or nutrient deprivation, cells may struggle to obtain adequate oxygen or nutrients to support regular metabolic functions. In states of starvation, GAPDH can be modulated and suppressed, subsequently obstructing the glycolytic pathway. This intervention, known as starvation therapy, diminishes a cell’s reliance on glucose, compelling it to seek alternative metabolic pathways like fatty acid oxidation and autophagy. Jiang et al. prepared a two-dimensional MOF Gd-TCPP nanochip (Gd-TCPP-NC) coordinated with NC and combined SDT with starvation therapy using the thiol-ene click reaction to improve the efficiency of tumor treatment [117]. The results of the study showed that Gd-TCPP-NC could consume GSH and inhibit the glycolytic activity of GAPDH in the absence of oxygen in in vitro experiments, thus improving the therapeutic efficacy of starvation therapy, and the results of in vivo experiments also illustrated that the use of the thiol-ene click reaction by Gd-TCPP-NC to kill the tumors could improve the efficiency of the treatment of tumors.

### 5.6. Combined Treatment with SDT and Immunotherapy

**Figure 7:**
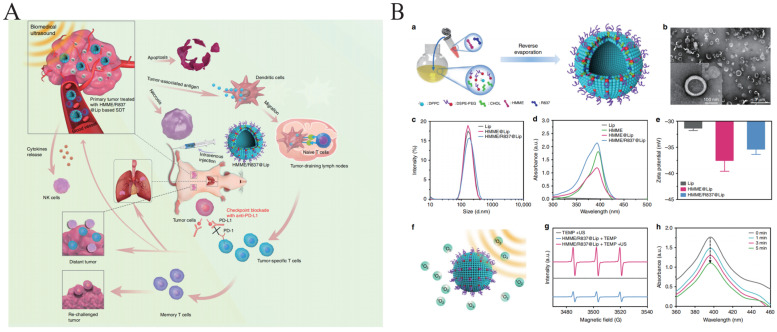
Combined treatment with SDT and immunotherapy. (**A**) Antitumor immune responses induced by SDT in combination with immunoadjuvant-containing nano photosensitizers and checkpoint blockers. (**B**) Synthesis and characterizations of the composite nanosonosensitisers. (**a**) Schematic illustration of the construction of HMME/R837@Lipnanosonosensitisers and their microstructures; (**b**) TEM image showing the quasi-spherical morphology of HMME/R837@Lip with high dispersity; (**c**) hydrodynamic diameters of HMME/R837@Lip nanosonosensitisers in PBS as measured by DLS; (**d**) UV-vis absorbance spectra of Lip, HMME, HMME@Lip and HMME/R837@Lip, indicating the successful encapsulation of HMME into the nano-liposome; (**e**) zeta potential of Lip, HMME@Lip and HMME/R837@Lip; (**f**) scheme of US-triggered ^1^O_2_ production as assisted by HMME/R837@Lip; (**g**) ESR spectra of HMME/R837@Lip with or without US treatment; (**h**) time-dependent DPBF absorption spectra in the presence of HMME/R837@Lip under US irradiation for varied durations. Reproduced with permission from Ref. [104].

SDT has the capability to release numerous TAAs, thereby stimulating the body’s immune system to mount an immune response against tumor cells. Furthermore, SDT plays a crucial role in enhancing the TME, boosting the infiltration and activity of immune cells, consequently augmenting the efficacy of immunotherapy. Additionally, SDT has the potential to induce apoptosis and necrosis in tumor cells, leading to the liberation of additional tumor antigens, which further enhances the cytotoxic effect of immune cells. These combined effects contribute significantly to improved treatment outcomes. The programmed cell death-1/programmed cell death-Ligand 1 (PD-1/PD-L1) signaling pathway serves as a critical mechanism through which tumor cells evade immune surveillance. Immunotherapies targeting the PD-1/PD-L1 signaling pathway have emerged as a leading strategy for treating a diverse array of tumors, aiming to disrupt this immune evasion mechanism and enhance the immune response against cancer cells [118]. However, immunotherapy does not have significant therapeutic effects on all patients, and adverse reactions may occur among some patients. By combining SDT with immunotherapy, there is a promising opportunity to overcome the constraints and limitations of immunotherapy. This synergistic approach has the potential to enhance treatment outcomes significantly, mitigating adverse reactions while improving therapeutic effectiveness. Yue et al. prepared a nanoplatform (HMME/R837@Lip) of liposome-encapsulated HMME and the immune adjuvant imiquimod (R837) (Figure 7) [119]. As shown through in vivo and in vitro experiments, anti-PD-L1 checkpoint blockade contributes to enhanced immune response, and HMME/R837@Lip-mediated SDT in combination with anti-PD-L1 checkpoint blockade immunotherapy was able to halt primary tumor progression while providing long-term immune memory to prevent tumor recurrence. This combination therapy strategy utilizes antigens released after tumor cell death to stimulate the immune system in combination with immunotherapy and checkpoint blockade, demonstrating potential synergistic effects and offering new ideas and possibilities for tumor treatment.

TAM plays a crucial role in the TME, influencing the uptake and distribution of tumor NPs [120]. Most monocytes differentiate into TAM in response to tumor cytokine stimulation, shifting between M1 and M2. The term M1 refers to pro-inflammatory and antitumorigenic macrophages, whereas the term M2 refers to anti-inflammatory and tumorigenic macrophages. M2 macrophages suppress antitumor immunity by secreting several anti-inflammatory factors, such as IL-4, IL-10, and IL-13, as well as inducing apoptosis. In contrast, M1 promotes antitumor immunity by secreting pro-inflammatory cytokines such as IL-6, IL-12, IL-23, TNF-α, and by inducing matrix degradation. It was shown that nanovesicles derived from M1 macrophages could effectively target tumor tissues, repolarize M2 TAM to M1, and release pro-inflammatory cytokines, thereby activating antitumor immune responses [121]. Therefore, utilizing nanovesicles derived from M1 macrophages as nanocarriers presents a promising approach to precisely targeting tumors within the TME, guiding M2 TAM repolarization to M1, stimulating the immune system, and inhibiting tumor growth. This innovative strategy is anticipated to emerge as a critical avenue in tumor therapy, offering novel insights and methodologies that can enhance the efficacy of cancer treatment. Chen et al. encapsulated PLGA NPs on M1 macrophage membranes and loaded the acoustic sensitizer IR780 and catalase (CAT) to form an immune-enhancing nano-system (M1/PLGA@IR780/CAT NPs) [122]. M1/PLGA@IR780/CAT NPs can promote dendritic cell maturation via SDT and modulate the tumor immune microenvironment to induce tumor cell death. The combination therapy of this nano-system with anti-PD-L1 immune checkpoint blockade transforms an immunosuppressive TME into an immune-promoting environment that triggers a systemic antitumor immune response and robust immune memory. This combination therapy has multiple mechanisms of action, making it a very novel and promising strategy for tumor treatment. However, it is important to note that any new therapeutic approach needs to undergo rigorous clinical trials to validate its efficacy and safety before it can be applied in a clinic.

## 6. Clinical Applications and Limitations of SDT

SDT has demonstrated significant potential in clinical applications and is currently the focus of extensive research and exploration (Table 1). The clinical application of SDT encompasses several key aspects, which are noted in the following. Tumor therapy: SDT utilizes sound waves to induce the aggregation and rupturing of micro-vesicles within tumor tissues, leading to local mechanical destruction and thermal effects that result in apoptosis and necrosis of tumor cells. SDT offers advantages such as precise targeting, minimal trauma, and reusability and has emerged as a prominent research area in the field of tumor therapy. Tumor targeted therapy: SDT can be combined with targeted micro vesicles or nano-drug carriers to achieve precise treatment of tumor targets. This targeted therapy strategy can improve the therapeutic effect and reduce the damage to normal tissue. Image-guided treatment: By integrating SDT with image-guided technologies like US imaging, accurate tumor positioning and monitoring can be achieved, enhancing treatment precision and safety. Immunomodulatory effect: SDT has the capability to modulate the TME, facilitating the release of antigens, immune cell infiltration, and activation of the immune system. This immunomodulatory effect enhances the effectiveness of immunotherapy by boosting the body’s immune response against cancer cells.

SDT is clinically utilized in the treatment of various cancers, including those affecting the liver, breast, pancreas, and lungs. In a case report by Wang et al., the clinical outcomes of three patients with advanced refractory breast cancer who underwent SDT in combination with PDT were described [123]. The treatment involved the sublingual absorption of the acoustic sensitizer SF1 over 2 to 3 days, followed by irradiation of the tumor area or the entire body for 30 min 24 h after the final administration using a red LED lamp (wavelength 630 nm, power 20 Mw/cm^2^). This was succeeded by a 20 min irradiation of the tumor area for 3 consecutive days using a portable ultrasonic instrument (frequency 1 MHz, power 2.0 W/cm^2^). Subsequently, all three patients exhibited significant reductions in tumor size, with no noticeable effects on systemic vital organs following SDT and PDT. These findings suggest that the combined approach achieved a certain therapeutic effect on patients with advanced refractory breast cancer without exerting significant effects on systemic vital organs during treatment. In another case report, researchers documented the treatment of a patient with advanced breast cancer using SDT in combination with immunotherapy [124]. The patient presented with invasive ductal carcinoma and advanced disease with metastases to the right axilla, spine, and pleura, along with an ER^+^, PR^+^, and HER2^+^ status. Treatment options included SDT combined with immunotherapy, employing specific therapeutic agents such as 5-aminolevulinic-acid-modified Ce6 and exemestane. Following 19 US treatments, the patient’s tumors in the right axilla and pleura completely disappeared, and tumor markers rapidly decreased without serious side effects. These results indicate that SDT combined with immunotherapy yielded significant therapeutic outcomes for this patient and may hold promise for individuals with advanced breast cancer. However, these findings are based on a case report with a small sample size, underscoring the need for further clinical trials and case accumulation to validate the efficacy and safety of this therapy in treating advanced refractory breast cancer.

In addition to the findings, the combination of SDT with other therapies such as chemotherapy, PDT, and immunotherapy demonstrates potential synergistic effects in tumor treatment, offering promise for enhancing treatment success. Nonetheless, further clinical studies and validations are imperative to ascertain the specific role of SDT in treatment success [125]. When SDT is combined with other therapeutic modalities, several challenges and areas warranting additional research may emerge, including the following. Therapeutic mechanisms: The combined use of diverse therapeutic modalities could impact tumor cell growth, metastasis, and drug resistance. However, a comprehensive exploration of the interaction mechanisms between these modalities is essential [126]. Side effects and safety: the utilization of different treatment modalities may elevate the risk of toxic side effects, underscoring the importance of rigorously evaluating the safety and tolerability of combination therapy [7]. Individualized treatment: Tailoring individualized combination treatment plans is crucial for different tumor types and stages. These plans should be adjusted based on the unique conditions of each patient [98,127]. Clinical efficacy assessment: the accurate assessment of the efficacy, prognosis, and survival rates associated with SDT in combination with other treatment modalities necessitates the development of a scientific assessment framework and the establishment of standardized indicators [128]. Addressing these challenges through further research and clinical trials will be instrumental in elucidating the full potential and optimizing the efficacy of SDT in combination therapies for cancer treatment.

**Table 1:** Clinical applications of SDT for the treatment of tumors.

Sonosensitizers	Cancer Type	Treatments	Result	Refs.
Ce6	breast cancer	macrophage-activating factor + SDT + hormone therapy	Complete resolution of pleural effusion and intrapleural nodular tumors without serious adverse effects	[124]
Two new chlorophyll derivatives	breast cancer	SDT + PDT	Almost no toxicity, and may dramatically enhance chemo efficacy in some refractory advanced breast cancer cases	[129]
Sonoflora 1™	breast cancer	SDT	Patients with advanced cancer were treated safely and effectively	[130]
Sonoflora 1™	breast cancer	SDT + PDT	All patients had significant partial or complete responses	[123]
5-aminolevulinic acid	Diffuse intrinsic pontine glioma	SDT	Cohort 1 was completed without dose-limiting toxicity	[131]

## 7. Future Perspectives and Conclusions 

SDT utilizing nanomaterials has emerged as an innovative approach in tumor treatment, garnering increasing attention. By leveraging the interaction of sound waves and micro-foamers, SDT can selectively destroy tumor tissues locally, offering advantages such as precise targeting, minimal trauma, reusability, and promising clinical applicability [132]. Based on existing research findings and clinical practice, the following conclusions can be made: effective tumor destruction—SDT can effectively eradicate tumor tissues and impede tumor growth and proliferation, thereby achieving therapeutic efficacy; penetration depth and conductivity—SDT exhibits favorable penetration depth and conductivity, enabling comprehensive coverage and treatment of tumor tissues; immune function enhancement—SDT has the potential to enhance the body’s immune function by eliciting an immune response, offering an adjunct role for immunotherapy; and synergistic effects in combination therapy—the concurrent use of SDT with other therapeutic modalities, such as PDT, chemotherapy, and immunotherapy, can yield synergistic enhancements, leading to improved treatment success rates. However, it is important to note that further research and validation are still necessary to gain a deeper understanding of the mechanism and specific role of SDT. In the context of combination therapy, more exploration is needed regarding the interaction between different treatment modalities, including assessing their safety, tolerability, and developing individualized treatment regimens for different tumor types and stages. Additionally, establishing a scientific efficacy assessment system with standardized indicators is crucial to accurately evaluate effectiveness, prognosis, survival rates, and other relevant factors when SDT is combined with other treatment modalities. Looking ahead, we anticipate that through continued clinical research and practice, we will gain further insights into the mechanism of action and efficacy assessment system of SDT. This will enable us to refine combined treatment strategies and personalized treatment plans, offering improved treatment options and renewed hope for patients. Furthermore, by integrating emerging technological means and treatment concepts such as nanotechnology, gene editing, artificial intelligence, and others, we can expand the application scope of SDT in tumor treatment and enhance treatment precision and personalization.

This review provides an overview of the principles, mechanisms, and combination therapies involving SDT while delving into the advantages and prospects of SDT in tumor therapy from various angles. Firstly, concerning the principles and mechanisms of SDT, this review elucidates how SDT utilizes sound waves or US waves to trigger nanomaterials for targeted drug release in tumor therapy. It further explores the synergistic treatment strategies of SDT, including its combined application with PTT, chemotherapy, and other therapeutic modalities to enhance therapeutic outcomes. Secondly, focusing on the selection and design of nanomaterials, this review examines the utilization of various types of nanomaterials in SDT and delineates the strengths and weaknesses of different nanostructures. Lastly, regarding the clinical applications and future trajectory of SDT, this review underscores the immense potential and promising outlook of this approach. While SDT is currently in the laboratory-research phase, it has already exhibited notable efficacy in tumor therapy. Moving forward, a deeper understanding of the advantages and limitations of SDT can be attained through continued research and clinical implementation, paving the way for its enhanced application in real-world clinical settings.

In conclusion, the utilization of acoustic dynamic therapy based on nanomaterials in tumor therapy holds significant importance and offers promising prospects. As science and technology continue to advance, alongside in-depth clinical research, we anticipate ongoing innovation and optimization of SDT in the future. We firmly believe that SDT will emerge as a crucial modality in the realm of tumor treatment, offering expanded treatment options and renewed hope for patients.

## Abbreviations

Ag	silver
AIPH	2,2-azobis[2-(2-imidazolin-2-yl) propane] dihydrochloride
ATP	adenosine triphosphate
BSO	buthionine sulfoximine
CAT	catalase
Ce6	chlorin e6
Chol	cholesterol
CRT	calreticulin
DAMPs	damage-associated molecular patterns
DC	dendric cell
DMDD	2-dodecyl-6-methoxycyclohexa-2,5-diene-1,4-dione
DNA	deoxyribonucleic acid
DOX	doxorubicin
DP	double positive cell
DTX	docetaxel
EPR	enhanced permeability and retention
ER	estrogen receptor
GAPDH	glyceraldehyde-3-phosphate dehydrogenase
GNRs	graphene nanoribbons
GOx	glucose oxidase
GSH	glutathione
GSNO	s-nitrosoglutathione
HA	hyaluronic acid
Hb	hemoglobin
HER2	human epidermal growth factor receptor-2
HMGB1	high mobility group protein 1
HMME	hematoporphyrin monomethyl ether
HNSs	hollow nanoshells
H_2_O_2_	hydrogen peroxide
HPB	hollow mesoporous Prussian blue
HSA	human serum albumin
HSP	heat shock protein
ICD	immunogenic cell death
IL	interleukin
MnO_2_	manganese dioxide
MnPcS	manganese phthalocyanine
MnTEPP	manganese porphyrin nanoparticles
MOFs	metal-organic frameworks
MPTP	mitochondrial permeability transition pore
NADPH	nicotinamide adenine dinucleotide phosphate
NB	nanobubble
NC	nanochip
NIR	near infrared light
NPs	nanoparticles
NSs	nanosheets
O_2_	oxygen
^1^O_2_	single-linear oxygen
O^2−^	superoxide anion radicals
·OH	hydroxyl radicals
ONOO	peroxynitrite
PD-1	programmed cell death-1
PD-L1	programmed cell death-Ligand 1
PDA	polydopamine
PDT	photodynamic therapy
PEG	polyethylene glycol
PFH	perfluoro hexane
PIC	piceatannol
PLGA	polylactic-co-glycolic acid
PR	progesterone receptor
PTA	photothermotransduction agent
PTT	photothermal therapy
PTX	paclitaxel
PVP	polyvinylpyrrolidone
RBC	red blood cell
rGO	reduced graphene oxide
ROS	reactive oxygen species
SDT	sonodynamic therapy
SL	sonoluminescence
SM	manganese porphyrin
SMA	manganese porphyrin-HAuCl4
SMAH	manganese porphyrin-HAuCl4-HA
TAAs	tumor-associated antigens
TAM	tumor-associated macrophages
TAPP	tetra-(4-aminophenyl) porphyrin
TiO_2_	titanium dioxide
TME	tumor microenvironment
TNF-α	tumor necrosis factor-α
TPP	triphenylphosphine
TPZ	tirapamide
TTP	4-methylphenylporphyrin
US	ultrasound
VEGF	vascular endothelial growth factor
